# Cotton Breeding in Australia: Meeting the Challenges of the 21st Century

**DOI:** 10.3389/fpls.2022.904131

**Published:** 2022-05-13

**Authors:** Warren C. Conaty, Katrina J. Broughton, Lucy M. Egan, Xiaoqing Li, Zitong Li, Shiming Liu, Danny J. Llewellyn, Colleen P. MacMillan, Philippe Moncuquet, Vivien Rolland, Brett Ross, Demi Sargent, Qian-Hao Zhu, Filomena A. Pettolino, Warwick N. Stiller

**Affiliations:** ^1^CSIRO Agriculture and Food, Narrabri, NSW, Australia; ^2^CSIRO Agriculture and Food, Canberra, ACT, Australia; ^3^Cotton Seed Distributors Ltd., Wee Waa, NSW, Australia; ^4^Hawkesbury Institute for the Environment, Western Sydney University, Richmond, NSW, Australia

**Keywords:** cotton, plant breeding, genomic selection (GS), gene editing, phenomics, GM traits, panomics, gene based breeding

## Abstract

The Commonwealth Scientific and Industrial Research Organisation (CSIRO) cotton breeding program is the sole breeding effort for cotton in Australia, developing high performing cultivars for the local industry which is worth∼AU$3 billion per annum. The program is supported by Cotton Breeding Australia, a Joint Venture between CSIRO and the program’s commercial partner, Cotton Seed Distributors Ltd. (CSD). While the Australian industry is the focus, CSIRO cultivars have global impact in North America, South America, and Europe. The program is unique compared with many other public and commercial breeding programs because it focuses on diverse and integrated research with commercial outcomes. It represents the full research pipeline, supporting extensive long-term fundamental molecular research; native and genetically modified (GM) trait development; germplasm enhancement focused on yield and fiber quality improvements; integration of third-party GM traits; all culminating in the release of new commercial cultivars. This review presents evidence of past breeding successes and outlines current breeding efforts, in the areas of yield and fiber quality improvement, as well as the development of germplasm that is resistant to pests, diseases and abiotic stressors. The success of the program is based on the development of superior germplasm largely through field phenotyping, together with strong commercial partnerships with CSD and Bayer CropScience. These relationships assist in having a shared focus and ensuring commercial impact is maintained, while also providing access to markets, traits, and technology. The historical successes, current foci and future requirements of the CSIRO cotton breeding program have been used to develop a framework designed to augment our breeding system for the future. This will focus on utilizing emerging technologies from the genome to phenome, as well as a panomics approach with data management and integration to develop, test and incorporate new technologies into a breeding program. In addition to streamlining the breeding pipeline for increased genetic gain, this technology will increase the speed of trait and marker identification for use in genome editing, genomic selection and molecular assisted breeding, ultimately producing novel germplasm that will meet the coming challenges of the 21st Century.

## Introduction

### Background and History of the Commonwealth Scientific and Industrial Research Organisation Cotton Breeding Program

**FIGURE 1:**
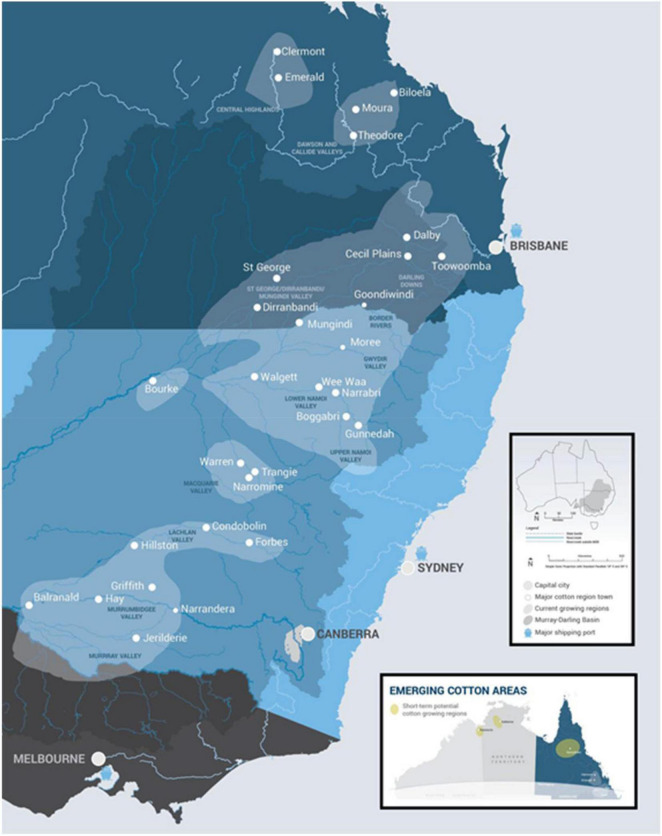
The Australian cotton growing regions extend from latitudes of –36° (Murray Valley) to –22° (Central Queensland). Source: Cotton Australia Ltd.

Although cotton has been grown in Australia since the late 1700’s, the modern Australian cotton industry was established in the early 1960’s when high-input irrigated cotton growing commenced in three States: New South Wales, Queensland, and Western Australia. The Western Australian industry, situated on the Ord River in the tropical north of the State, ceased production in 1974 due to the development of insecticide-resistant pests (Hearn, 1975). Production in the two eastern States has increased since 1960 from near-zero to its peak of over five million bales in 2012. The environment of the primary production areas of the eastern States ranges from sub-tropical to temperate/Mediterranean climates (i.e., from latitudes of –22° to –36°). Recently, production has begun to expand back into tropical regions (–18° to –14°) (Figure 1). The 10-year average amount of cotton produced annually is around 3.4 million bales (772,000 tons of lint) with the variability usually associated with availability of irrigation water.

United States cultivars were exclusively grown during the 1960’s and 1970’s (Low, 1974). During this period, breeding programs were established by CSIRO at Griffith (–36°) in southern NSW, New South Wales Department of Primary Industry (NSW DPI) at Narrabri (–30°) in northern NSW, Qld Department of Primary Industry (Qld DPI) at Biloela (–22°) in central Queensland and CSIRO at Kununurra on the Ord River (–15°) in Western Australia. These widely separated programs had different goals: the Griffith program, situated at the southernmost cotton growing region, was focused on very early maturity; NSW DPI’s on Verticillium wilt tolerance; Qld DPI’s on resistance to bollworms via raising gossypol level; while under tropical far northern Australian conditions, CSIRO sought to capitalize on apparent high levels of heterosis occurring between African and American Upland cultivar crosses (Thomson, 1971). After the failure of the industry in the Ord River and difficulties in establishing the industry in Southern NSW, CSIRO established the CSIRO Cotton Research Unit at Narrabri in 1972. The Griffith and Ord River programs were transferred to Narrabri and shortly thereafter both the NSW DPI and Qld DPI programs were closed. The primary focus of this new breeding program was developing full-season cultivars for the main Australian cotton growing areas, together with improving fiber quality attributes, particularly fiber strength and disease resistance. Cotton Research and Development Corporation (CRDC) was a major investor in the CSIRO Plant Breeding Program from 1990 to 2007, investing $46 million on behalf of growers.

CSIRO and CSD have been working together developing and commercializing cotton cultivars since 1971 and have jointly released over 116 cultivars during this period. In 2007, Cotton Breeding Australia (CBA) was formed as a joint venture between CSIRO and CSD. It is a targeted research fund which facilitates the research and development of future cotton cultivars for Australian growers. It is focused on the future needs and challenges for cotton production in Australia and since 2007 has invested over $146.47 million (as of June 2021) toward these research activities. The management structure for CBA consists of a Management Committee and a Scientific Committee. The Management Committee, with both CSIRO and CSD members, are responsible for the overall management, operation, and performance of the activities. The Scientific Committee, with both CSD and CSIRO members, as well as nominees from the Australian cotton industry bodies – Cotton Research and Development Corporation and Cotton Australia, collectively oversee the research activities and keep the Management Committee informed. The current program structure and operations provide the framework for the research and development as well as commercial cultivar delivery.

### Current Structure of Program

**FIGURE 2:**
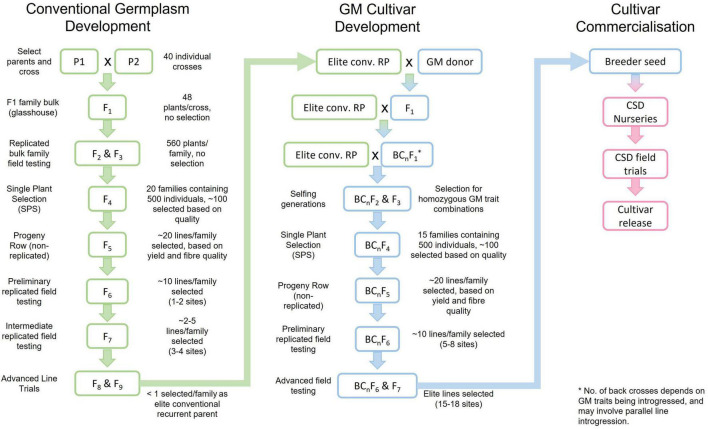
The breeding process used by the Commonwealth Scientific and Industrial Research Organisation (CSIRO) and Cotton Seed Distributors Ltd. (CSD) to develop conventional germplasm and introgress required GM traits, leading to cultivar commercialization. This is a simplified exemplar figure but captures the basic steps in the process. P1 and P2, Parent 1 and Parent 2; F, Filial generation; conv., conventional; RP, recurrent parent; GM, genetically modified; BC, back cross.

The strategies used in the CSIRO breeding program have continued to evolve over the years but have generally followed classical plant breeding methods based on field phenotypic selection. Although the Australian cotton industry exclusively grows cultivars with genetically modified (GM) traits for pest and weed management, significant resources are dedicated to the conventional germplasm enhancement program. This is where the improvements in yield, fiber quality, host plant resistance and regional adaptation are developed. The result is essentially a conventional breeding program in parallel to a GM trait introgression and breeding program (Figure 2). Selection of parents for hybridisation is a very important stage and while the introduction of diverse germplasm is always of high priority, it is more important to have high performing, well adapted parents. Originally germplasm from many sources were used and some of most successful parents were from Arizona, Mississippi, Texas, New Mexico, and Russia. Most parents used today are proprietary germplasm, but lines are continually introduced to our program from other breeding programs.

In the conventional breeding process, single plant selection commences at the F4 stage and large populations are evaluated (∼500 individuals). Characteristics with high heritability such as morphology (e.g., leaf shape, hair) and disease resistance [mainly bacterial blight (BB)] are used to initially screen the population. Visual selection culls few lines at this stage and large numbers of plants are individually harvested and ginned. Selection occurs for lint percentage and the fiber of the selected plants is evaluated for quality on a Spinlab High Volume Instrument (HVI) (Uster Technologies, AG, Uster, Switzerland). Selection is carried out for strength, length, length uniformity, extension and micronaire. In the following season a single progeny row is grown from each selected plant. Selection is based on disease resistance, leaf shape, hair, and plant type, as well as machine harvested yield, lint percent and fiber quality.

In the following years the lines progress through a series of replicated trials, beginning with one or two sites. In all cases row and column designs are used and analysis carried out using mixed models to adjust yields for spatial effects (Liu S. M. et al., 2015). To reduce the effects of genotype-to-genotype competition, trials use three or four row plots with the center rows harvested (Luckett et al., 1992). From preliminary trials, data is collected on morphology, disease resistance, plant habit, yield, and fiber quality. In more advanced trials similar data is collected but at up to five or six sites. These sites are chosen to cover the target production systems and growing regions. Aggressive selection for yield in segregating populations in environments with high potential yield is an important component of the breeding process. Most sites are located on commercial farms and the support of cotton growers is a significant component of the breeding program. This strategy captures current crop management practices used across the industry that the cultivars are developed for.

The process of developing cultivars containing GM traits follows from the identification of high performing conventional lines (Figure 2). The GM traits are incorporated using a standard backcrossing (BC) process (e.g., Priyadarshan, 2019). The number of BCs used is dependent upon the donor parent’s performance for agronomic characteristics, but two to four are common. From the conclusion of the BC process, an approach similar to the conventional breeding process is taken i.e., large populations of single plants, followed by progeny row and replicated testing to identify the best performing lines from within a backcrossed population. Other techniques such as marker assisted backcrossing (MABC) (e.g., Frisch and Melchinger, 2005) have been considered but not currently used. While MABC certainly has the potential to improve the efficiency of trait introgression, our aim is to develop the best possible performing line, not necessarily one identical to the recurrent parent. For this reason, we invest significant resources in selection for agronomic traits after the BC process is completed. This has often proved successful with a 2–4% yield increase over the recurrent parent (Stiller, 2022, personal communication, 11 February).

The final stage is handover of breeder seed to CSD for nursery seed increase, large scale pre-commercial testing and final cultivar release (Figure 2). Although it is ultimately the responsibility of the seed producer/seller to ensure that the products they sell to growers are fit for purpose and compliant with relevant consumer and regulatory laws, some responsibilities extend back into the breeding program to ensure that the Breeder Seed provided is of the highest quality. Such a requirement has always existed for non-GM (native) traits, especially visible traits like leaf type (okra vs. normal leaf) and has required some manual culling of “off-types” during the breeding pipeline, but the shift in Australia to GM cultivars has added new emphasis on Quality Assurance (QA) and Quality Control (QC). The aim is to avert any reputational or even legal or financial damage that could result from unapproved GM traits being unintentionally released to our seed partner and then to growers or released GM cultivars failing to live up to contractually specified purity or efficacy standards or compliance with international regulatory frameworks.

To manage these risks our breeding program has established a set of operational procedures to maximize genetic purity and minimize GM trait contamination. These procedures are also matched by CSD, to ensure commercial seed remains of high purity during the few years of seed increase prior to sale. During both conventional breeding and GM trait introgression, for example, every plant used in crossing is sampled and genotyped for a range of GM and native traits for which we have molecular markers (discussed in other sections) and then again at the F_2_ generation. This process is designed to select homozygous individuals for traits of interest and identify potential errors in the genotypes of the crossing parents. In all subsequent steps of increase and selection, random sampling is carried out to ensure purity, especially at the time of the hand-over of Breeder Seed (often F_6_ or F_7_) to CSD. Our procedures have ensured strict germplasm stewardship. As advanced lines must still be grown in the field to generate sufficient seed, they are unavoidably exposed to pollen from commercial crops growing nearby so some material can still fail purity testing. Those lines are either discarded or go back into a cycle of cleaning (every plant in the plot is genotyped and any off types removed) before subsequent seed increase. These QA and QC procedures are a considerable investment, but implementation is essential to ensure the entire cultivar development process meets commercial and legal obligations.

## Yield Progress

**FIGURE 3:**
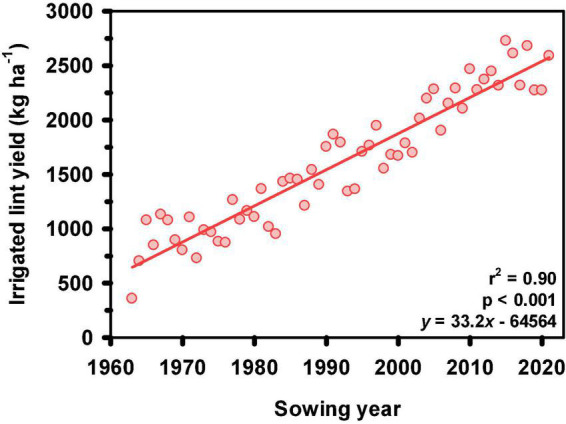
Average Australian irrigated cotton industry yield (kg [lint] ha^–1^) from 1962 to 2021. Source: The Australian Cottongrower Cotton Yearbook.

The Australian cotton industry continues to be of envy in the cotton world for its ongoing yield progress and maintenance of the highest yield (Constable and Bange, 2015). In the early 1960’s, when modern cotton production commenced in Australia, industry average yields were less than 1000 kg lint ha^–1^ for fully irrigated crops. Currently, this figure has more than doubled to just under 2600 kg lint ha^–1^ (Figure 3; Thomson, 1979; Constable and Bange, 2015). Rainfed cotton production area is variable and highly dependent on seasonal conditions and commodity prices, ranging from 5 to 30% of the total area. Yields are also variable, largely influenced by in-crop rainfall. Nevertheless, the average yield of rainfed produced cotton has also increased (Constable and Bange, 2015).

Yield progress is due to the persistent efforts of the Australian cotton industry around research and extension of new technologies. The technologies comprise improved cultivars, better crop management strategies with respect to disease, pest and weed control, irrigation and fertilizer application, crop rotation, tillage and the adoption of GMO traits for pest and weed control (Constable, 1998, 2004; Constable et al., 2011; Constable and Bange, 2015). Of these, the release and adoption of new cultivars has been the primary driver (Constable et al., 2001; Liu et al., 2013; Rochester and Constable, 2015; Conaty and Constable, 2020).

Harvestable yield is the ultimate measure of cultivar performance. When grown under similar crop management practices and target environments, yield progress can therefore be assumed to be a result of the contribution of a new cultivar (genetics), management and their interaction. An analysis of long-term breeding trial datasets of conventional cotton in the period of 1980 to 2009, the role of 23 mainstream cultivars released from the CSIRO cotton breeding program were assessed for yield increase. Yield gain was reported to range from 7.0 to 18.3 kg lint per ha per year, with the highest gain recorded in the latest 15-year period. By testing a set of the same conventional cultivars released from 1973 to 2006, Rochester and Constable (2015) reported yield increase of 28.8 kg lint per ha per year. Constable et al. (2011) reported yield gain of 26 kg lint per ha per year since 1963 until 2010, concluding similar yield gain was maintained when entering the GM era from 1996.

Liu et al. (2013) used yield estimates from a 30-year data set of ten CSIRO cultivars, splitting the dataset into early (1980–1994) and late 15-year periods (1995–2009). Analysis by linear mixed model demonstrated that in terms of yield progress, genetic improvement contributed the largest (48%), followed by management and the interaction of genetics × management, each contributing almost equally (24% vs. 28%, respectively). The findings are consistent with early similar work of which 45% of the contribution was due to cultivar while the remainder is from improved crop management, with 25% from soil and irrigation, 20% from insect control and 10% from disease management (Constable, 2004). This evidence not only confirms the importance of improved cultivars for yield increase, but also the importance of exploiting the synergistic response of cultivars to improved management practices by focusing breeding efforts on yield gains in modern cotton production systems.

More recently, Conaty and Constable (2020) assessed the yield progress of 10 cultivars significant to the CSIRO cotton breeding program, released between 1968 and 2012. With the aim of identifying opportunities for future yield progress, the primary physiological determinants of lint yield: total dry matter and harvest index; as well as the secondary determinates of reproductive dry matter allocation, lint percentage, boll size, boll number, light interception, carbon assimilation, leaf area index and light extinction coefficient were also assessed. Yield progress was measured at 16.1 kg lint ha^–1^ y^–1^ and it was identified that selection pressure resulted in improvements in total dry matter (TDM), harvest index (HI), lint percentage and carbon assimilation. While gains were made in these four parameters, further analysis identified that improvements in lint yield were largely driven by altering HI through increasing lint percentage. Future yield progress cannot be made through further increases in lint percentage as further partitioning of carbon to lint comes at the expense of resource supply to seed, ultimately resulting in a reduction in seed weight which may result in reduced crop establishment and seedling vigor. Thus, avenues for future gains in lint yield will require the concurrent maintenance of HI while producing larger plants with more fruiting branches that capture more incident radiation with increased efficiency.

### Genetically Modified Approaches to Yield Enhancement

Over the last 20 years CSIRO has invested heavily in crop genomic capabilities including in cotton. CSIRO pioneered the development of printed cDNA glass microarrays to allow genome-wide gene expression analysis in cotton (Wu et al., 2006) and were early adopters of Next Generation RNA sequencing technologies in cotton. These genomic tools, combined with some unique germplasm resources, were instrumental in discovery and analysis of some of the key transcription factors (GhMYB25, GhMYB25-Like and GhHD-1) that regulate early events in the formation of the fiber initials on the surface of the seed (Machado et al., 2009; Walford et al., 2011, 2012). Reducing the expression of these regulatory factors using RNA interference (RNAi) in transgenic cotton demonstrated that GhMYB25-Like was critical for proper fiber initiation (reduced expression resulting in a fibreless seed phenotype). The other two factors were likely to be downstream of GhMYB25-Like and are needed for regulation of the timing and elongation of fiber initials (as well as being involved in leaf and stem trichome development). Interestingly, Scanning Electron Microscopy indicated that over-expression of any of these three transcription factors in transgenic cotton, led to an increase in the number of epidermal cells on the seed that initiated as fibers on glasshouse grown plants, and in some cases to hairier plants due to a greater production of leaf and stem trichomes. It was hypothesized that the greater proportion of initiated fibers might translate into greater numbers of mature fibers per seed, a significant component of overall fiber yield.

Crosses were initiated to bring together different combinations of the transgenes over-expressing the three identified transcription factors and the single and multiple homozygous transgene combination lines were tested in the field over two seasons (Liu S. M. et al., 2020). The results were both promising and disappointing; there were a small number of individual lines with single or double combinations of transgenes that outperformed their controls by up to 20%, but this was never consistent by transgene or combination. There also appeared to be a fine balance needed in gene expression as triple gene combinations were significantly worse than their non-transgenic controls. None of the lines with enhanced yield outperformed locally adapted cultivars as they were introduced into the genetic background of a transformable but unadapted cultivar from the U.S. They would require extensive backcrossing into elite Australian lines prior to evaluation and it would be difficult to prove that any improved performance was a consequence of transgene expression and not due to somaclonal variation from tissue culture, or just reassortment of native alleles in the different backgrounds. Ideally, transgenes should be re-evaluated in an adapted Australian cultivar directly.

## Fiber Quality Progress

**FIGURE 4:**
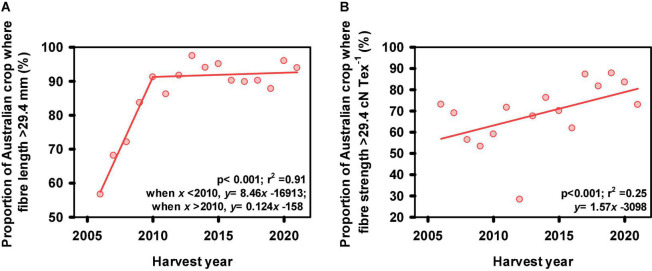
**(A)** The proportion of the Australian cotton crop where fiber length exceeded 29.4 mm (37 32nds of an inch) as a function of harvest year. Note the rapid increase in fiber length (8.5% yr^–1^ increase) between 2006 and 2010 as breeding efforts were focused to improve length and the maintenance of fiber length since 2010; **(B)** The proportion of the Australian cotton crop where fiber strength exceeded 29.4 cN Tex^–1^ (30 g Tex^–1^) as a function of harvest year. Note, data represents ∼90% Australian cotton crop. Source: Australian Cotton Shippers Association.

During the first two decades of the modern Australian cotton industry, the fiber produced had relatively poor strength and length. In the early 1980’s, the industry made the decision to switch completely to higher fiber strength cultivars while concurrently improving fiber length. This was designed to facilitate global marketing, as most of the Australian crop was and still is, exported. The breeding program has continued that standard, with each successive new cultivar aimed at maintaining or improving fiber properties. This strategy has proved very successful (Figure 4), with Australian export cotton now enjoying a reputation for excellent fiber properties, approaching the high-quality types such as San Joaquin Valley (SJV) from California. This progress has been due to intensive selection for all fiber quality traits from as early as the SPS breeding stage using HVI properties (Figure 2).

It is widely accepted that cotton fiber yield and quality are negatively associated, with the most significant association between strength and yield (Al-Jibouri et al., 1958; Meredith and Bridge, 1971; Clement et al., 2012). As such, significant research has been dedicated to understanding these associations and developing breeding strategies to break these associations. Clement et al. (2012) confirmed that in the CSIRO cotton breeding program a negative association still exists between fiber quality and yield. It was concluded that these negative associations are not due to photosynthetic capacity (Clement et al., 2013) and that the breaking of this linkage is one possible component of the progress that has been made in decreasing this association (Clement et al., 2012). Strategies were developed to identify parents as well as early generation selection for identifying better combinations of fiber quality and yield potential. Briefly these strategies focus on selecting locally adapted high strength parents to ensure improvements in both yield and strength, as well as other quality traits (Koebernick et al., 2019), and selecting a higher proportion of high yielding test lines (top ∼30%) in early generation testing (i.e., progeny row stage, see Figure 2) relative to fiber quality selections (top ∼10%) (Clement et al., 2015). This is followed by selecting the best yield and fiber quality combinations in subsequent replicated testing. These strategies are now routinely deployed in the CSIRO cotton breeding program, and additional strategies are under development.

## Disease Resistance

Disease is responsible for significant and widespread losses to cotton production in Australia. Reducing the impact of major pests and diseases through host plant resistance represents an effective way to realize the true yield potential of elite cultivars. Resistant germplasm is the most effective long-term means for minimizing yield losses and the deleterious environmental impacts of using other chemical control measures. Australia has had a long and successful history of tackling important diseases through the breeding of cultivars with increased genetic resistance.

### Bacterial Blight

In the early years of the modern cotton industry, BB of cotton caused by a bacterium *Xanthomonas citri* subsp. *malvacearum (Xcm)* was the most destructive and widespread disease, with around 20% of all bolls affected (Allen and West, 1987). A combined effort of seed production protocols to ensure blight-free planting seed and the breeding of cultivars that were immune to blight, controlled the disease to such an extent that is no longer found in Australian production systems. The single dominant BB resistance gene (Rungis et al., 2002) that is routinely bred into all Australian cultivars has been found to be strong, durable, and effective against all known races of BB found in Australia.

### Cotton Bunchy Top

Cotton bunchy top (CBT) disease is an aphid transmitted Polerovirus disease that results in severely stunted plant growth, that was first recorded in the late 1990s when a severe outbreak occurred resulting in heavy economic losses. Initially nearly all Australian cotton cultivars were identified as susceptible to the disease, with the only available control the use of insecticide against the vector the cotton aphid (*Aphis gossypii*). Molecular analysis of resistance cultivars identified the dominant resistance gene to a small interval (Ellis et al., 2016) resulting in the development of marker-assisted selection of CBT resistance with the first resistant cultivar (Sicot 620) released in 2019.

### Verticillium Wilt

Verticillium wilt (VW) caused by *Verticillium dahliae* has been an important disease in Australia for many years, and unlike many countries which only have the virulent defoliating pathotype, Australia has both highly virulent non-defoliating and defoliating pathotypes (Schnathorst and Evans, 1971; Chapman et al., 2016). The first CSIRO cultivar with significant resistance to this fungal pathogen was Sicala V-1 in 1991, which showed greatly reduced levels of infection, less severe symptoms, and higher yields. The majority of cultivars now grown commercially have relatively strong resistance to non-defoliating VW, but significant losses can still occur in seasonal conditions that favor the disease. Breeding for higher levels of resistance to non-defoliating and defoliating pathotypes is a major focus for ongoing research. Advancements in screening and selecting for VW resistance have been facilitated by both bioassay and field screening. Often, large-scale bioassay screening identifies resistance, and the genotypes are field tested in the next cotton season. The development of resistant populations is heavily reliant on multi-site disease nurseries to expose the populations to different isolates of VW. However, the two pathotypes of VW are treated as two separate breeding targets.

### Fusarium Wilt

Fusarium wilt (FW) caused by *Fusarium oxysporum* f. sp. *vasinfectum* was identified in Australian cotton in the early 1990s (Kochman, 1995). The Australian isolate is indigenous (Kim et al., 2005) and differs from those found in other countries in that it is associated with alkaline clay soils and is virulent in the absence of nematodes (Colyer, 2003). The extreme virulence and persistence of this pathogen and its ability to be readily transported in soil, water or trash was a major concern to the industry as most commercially grown cultivars at the time were highly susceptible with production losses of virtually 100% reported in some fields. New cultivars with increased FW resistance were developed based on a systematic analysis of the levels of resistance within the CSIRO germplasm collection that included over 200 genotypes. In 2004 the cultivar Sicot F-1, with twice the resistance of the benchmark cultivar in 1994, was released. Consistent progress in breeding for improved FW resistance has continued, with initial sources of resistance derived from Indian and Chinese *G. hirsutum* parents, and more recently from *G. hirsutum* and *G. barbadense* landrace cottons. Although the impact of FW on yield has been significantly reduced, in seasons that favor the disease or in fields that have a high level of inoculum, the most resistant cultivar may still only have 10% of plants uninfected. Thus, FW remains a significant breeding challenge.

### Black Root Rot

**FIGURE 5:**
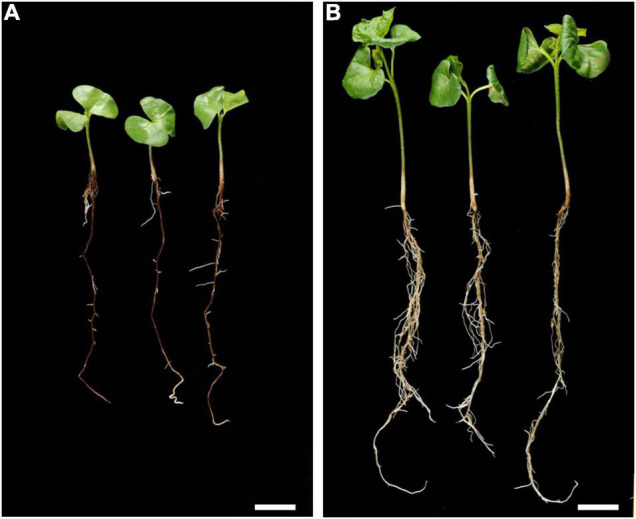
Parental phenotypes of the **(A)** BRR infected and susceptible YZ; and **(B)** the BRR infected and resistant BM13H infected seedlings 25 days after germination in soil with BRR spores. Bars = 2 cm. Adapted from Wilson et al. (2021) and is licensed under CC BY 4.0.

Black root rot (BRR) caused by *Berkleyomyces rouxiae* was first reported in Australian cotton in 1990 (Allen, 1990) and is considered a significant threat, especially in regions with shorter production seasons. Diseased cotton plants show stunted or slow growth early in the season compared to uninfected plants, causing delayed flowering or maturity that can result in up to a 46% decrease in seed cotton yield (Nehl et al., 2004). In addition to the direct effect of BRR infection, lesions caused by the fungus may facilitate infection by other cotton seedling pathogens. BRR resistance has not been found in any *G. hirsutum* or G. *barbadense* germplasm, so breeding for resistance must access secondary germplasm sources such as diploid cotton species. Resistance to BRR has been identified in *G. arboreum* (Figure 5) and the inheritance of resistance to BRR was evaluated in an F_6_ recombinant inbred population and shows a single semi-dominant locus conferring resistance that was fine mapped to a region on chromosome 1, containing ten genes including five putative resistance-like genes (Wilson et al., 2021). Although the use of secondary germplasm sources is a long and difficult process, even with modern molecular tools, field evaluations of germplasm with these sources of BRR resistance are currently undertaken.

## Host Plant Resistance to Insects and Arthropods

Cotton is vulnerable to a number of pests that impact fiber quality and yield (Matthews, 1989). Despite the success of integrated pest management (IPM) practices (Wilson et al., 2018), chemical control is still needed for the effective control of cotton pests. The implementation of GM cultivars for pest control has been successful throughout the industry. This success has been facilitated by constant monitoring of resistance development in the target insects and planting non-GM cotton to allow for the dilution of resistant alleles (Downes et al., 2017). However, pests not controlled by the GM traits have usually emerged as problems, requiring the development and use of resistant germplasm to target these newly emerging pests in order to maintain yields and reduce the quantity and number of chemicals applied to the crop (Trapero et al., 2016). The CSIRO cotton breeding program has seen success in host plant resistance (HPR) to insects and arthropods, targeting two-spotted spider mites and silverleaf whitefly (SLW) (Egan and Stiller, 2022).

### Silverleaf Whitefly

Silverleaf whitefly is an important pest to cotton (Miyazaki et al., 2013) found across all major cotton regions in Australia (Hall et al., 2012). SLW excrete sugars that cause honeydew contamination on the lint and result in a downgrade of quality and price received by the grower (Venugopal Rao et al., 1989; Hequet and Abidi, 2002). The control of SLW is expensive and difficult and will be reliant on HPR traits to be controlled. The okra leaf shape has been shown to provide morphological resistance to SLW. Cotton genotypes that are okra-leafed host fewer SLW than normal-leafed genotypes, as the okra leaf shape provides a less desirable environment due to the more open canopy (Butler et al., 1988; Chu et al., 2002). In addition to the okra-leaf trait, glabrous (no leaf hair) has also been identified as a constitutive morphological trait conferring resistance to SLW. Cotton genotypes with very smooth (glabrous) leaves harbor less SLW than moderately hairy leaves (Butter and Vir, 1989; Butler et al., 1991). Breeding efforts are now focused on incorporating both the okra leaf shape and the glabrous trait into elite backgrounds (Miyazaki et al., 2013).

### Two-Spotted Spider Mite

**FIGURE 6:**
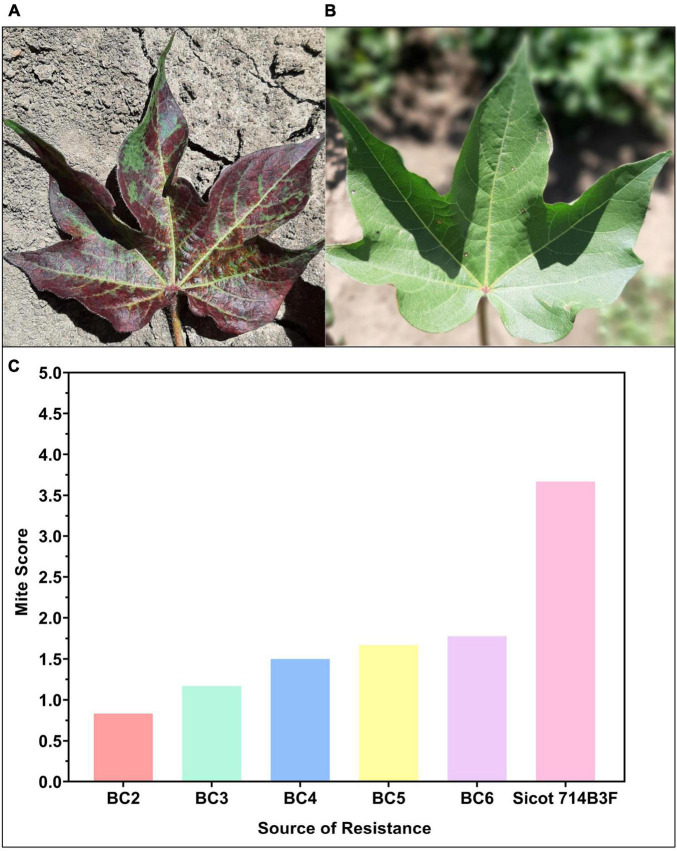
**(A)** Susceptible (Sicot 714B3F recurrent parent) and **(B)** resistant two-spotted spider mite cotton germplasm from the CSIRO cotton breeding program (Photos: Lucy Egan). **(C)** Progress of breeding mite resistant germplasm showing that as backcross (BC) generation number increases mite resistance scores have remained lower than the susceptible recurrent parent, Sicot 714B3F, and relatively stable. Data from C. Trapero, used with permission.

Two-spotted spider mite (*Tetranychus urticae* Koch) (TSSM) is a secondary pest of cotton (Miyazaki et al., 2012). TSSM are a sucking pest and ultimately reduce the photosynthetic capacity of the leaves by sucking out the contents of the mesophyll cells (Figure 6). Reduced yield and fiber quality are often observed from TSSM infestations (Wilson, 1993). Although TSSM is considered a secondary pest of cotton, the introduction of GM cotton cultivars has seen the prevalence and incidence increase across the industry (Wilson et al., 1998). The chemical control of TSSM is relatively expensive and can select for resistance in the mite populations. Exploiting host plant resistance traits to TSSM is a current breeding target for the program and novel germplasm with resistance is now part of the commercial breeding pipeline (Miyazaki et al., 2012, 2015). Our breeding efforts show that the incorporated TSSM resistance has remained high across consecutive backcross generations when compared to commercial controls (Figure 4).

**FIGURE 7:**
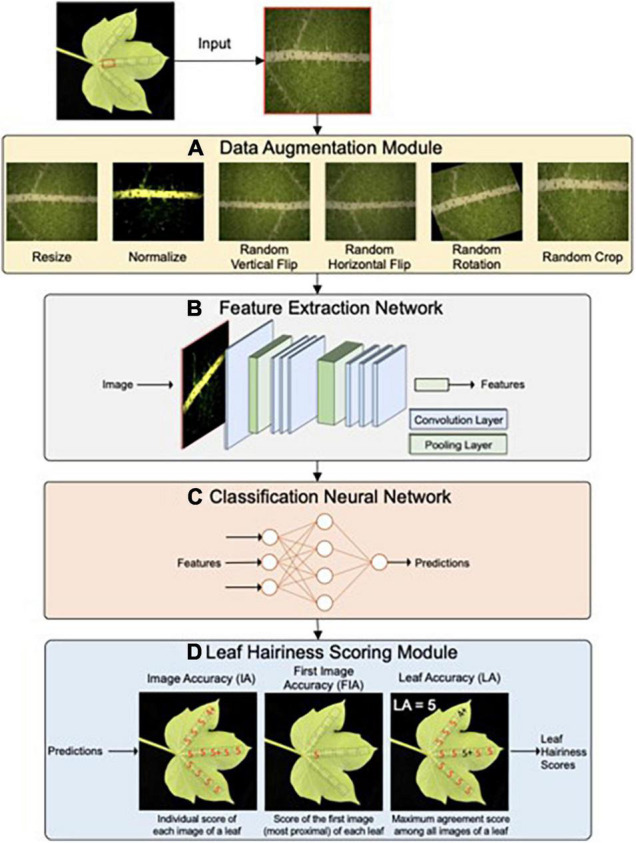
Network architecture of the HairNet deep learning model to score cotton leaf hairiness. HairNet consists of four main parts. First a leaf surface images are passed through a Data Augmentation module **(A)** that augments each image by applying a variety of image processing techniques. Processed images are then passed to a Feature Extraction Network **(B)** that extracts discriminative visual features from the image representation. Extracted visual features are then passed to a simple Classification Neural Network **(C)** that assigns each input image to a specific leaf hairiness score. Raw scores are then processed by the Leaf Hairiness Scoring module **(D)** which generates three accuracy metrics for scoring cotton leaf hairiness. Adapted from Rolland et al. (2022) and is licensed under CC BY 4.0.

## Abiotic Stress Resistance

Abiotic stressors are the primary cause of crop loss worldwide (Boyer, 1982), and despite the challenge of their genetic complexity and interactions with the environment are a foci of plant breeding efforts (Gilliham et al., 2017).

### Water Stress Tolerance

The future of the cotton industry and its potential to expand into additional rainfed summer cropping regions, requires the identification and development of cultivars that can remain productive despite periods of water stress. The initial focus of the CSIRO rainfed cotton breeding program was to target cultivar yield potential by undertaking early generation breeding and selection under fully irrigated conditions and evaluating advanced germplasm under rainfed conditions. However, in the mid-1990s a renewed focus on direct breeding and selection under water limited conditions was initiated. While this focus concluded that selection under dryland conditions would be beneficial and significant cultivar × water interactions occurred, this interaction varied with site; suggesting considerable environmental interactions in terms of rainfed performance with respect to amount and timing of rainfall (Stiller et al., 2004). It was also determined that under the variable rainfall environment that characterizes the Australian rainfed cotton production system, the phenotypic plasticity of later maturing cultivars provides a yield advantage (Stiller et al., 2004). As such, after a period of incorporating selection under rainfed environments, the program has again moved away from early and mid-generation evaluations and selection under rainfed conditions. However, due to the highly variable rainfall environment in the Australian cotton growing regions, the CSIRO cotton breeding program now employs a Managed Stress Environment protocol. This protocol applies irrigation water to rainfed breeding experiments in very dry years to better match our breeding environment with our testing environment (Conaty et al., 2018).

In addition to breeding and selecting for yield under water limited conditions, the CSIRO cotton breeding program has also invested research into identifying physiological traits conferring water stress tolerance and water use efficiency. Studies investigating leaf level gas exchange parameters and carbon isotope discrimination (Δ) concluded that these physiological traits are limited due to lower heritability than yield when measured under the same environmental conditions (Stiller et al., 2005). The use of canopy temperature from fixed infra-red sensors, a tractor-based phenotyping platform and from unmanned aerial vehicles (UAV) for the identification of water stress tolerant germplasm concluded that the measurement was not useful in a commercial cotton breeding program. This decision was based on issues with scalability, the inability to resolve genotype differences and lower heritability than yield. The pragmatic approach of testing technologies and discontinuing these where no commercial benefit can be realized is highlighted by research undertaken with respect to Δ and canopy temperature. Presently, more fundamental research is progressing to understand water conservation traits by the response of cotton water use (transpiration) to atmospheric (vapor pressure deficit, VPD) and soil water availability (fraction of transpirable soil water, FTSW). While Broughton and Conaty (2022) showed that there is genetic variation to the limiting transpiration at VPD trait, yield performance under limited water conditions is not improved in cultivars with the limiting transpiration trait at high VPD environments. Future research will explore the effect of the FTSW on transpiration and crop performance under limited water scenarios, as well as linking these traits and their physiology with the transcriptome and metabolome. This will then be used to identify a list of candidate genes that underpin water conservation traits that may be used to improve prediction accuracy of a rainfed genomic selection (GS) model.

### Heat Stress Tolerance

Despite cotton’s origin in warm, semiarid climates, efforts to maintain and improve cotton yields are expected to be hampered by high temperature stress (Bange et al., 2016). Subsequently, improving cotton’s thermotolerance has become an objective of the CSIRO cotton breeding program. Historically, research efforts were undertaken to screen diverse genotypes for heat tolerance, taking a multi-level approach from yield through to plant architecture, leaf-level gas exchange and cellular membrane integrity and biochemistry (Cottee et al., 2010). This research also developed a controlled environment screen which was scalable to the identification of parents for controlled crossing, but not useful in segregating breeding populations (Cottee et al., 2012). The molecular changes associated with the physiological performance and heat tolerance of cotton cultivars was also identified, with the aim of using this information to aid breeding for improved performance in warm and hot field environments (Cottee et al., 2013). More recently, Jaconis et al. (2021) refined a triphenyl tetrazolium chloride (TTC) based enzyme viability test following high-temperature stress to be used to identify vegetative heat tolerance phenotypes. Current research is assessing the potential gains in thermotolerance through concurrent selection of superior phenotypes based on this assay and yield performance in hot environments. Additional studies have also investigated the effect of high temperatures on pollen viability. These studies concluded that while pollen viability is affected by high temperatures (>39.5°C) and that there is genotypic variability in pollen thermotolerance, this genotypic variability has a limited effect on fruit retention as high fruit retention rates can be maintained at very low pollen viability. Furthermore, as cotton is indeterminate and produces many more flowers and pollen than required, in a breeding target environment characterized by short-term heat waves, pollen thermotolerance provides little advantage. It is concluded that carbon availability is the likely limitation on fruit retention under high temperature stress.

### Sodium Tolerance

Soil sodicity is one of the major soil constraints in Australian cotton production causing yield reduction of up to 20% (Rochester, 2010). The CSIRO cotton breeding program has developed research approaches to deliver sodicity tolerant cotton. Observations of differences in leaf Na concentration and K/Na ratio between *G. hirsutum* and *G. barbadense* germplasm led to the hypothesis that a leaf Na exclusion trait would secure better nutrition status of plants, and subsequently better nutrient use efficiency in sodic soils (Rathert, 1982; Liu S. et al., 2020). Liu S. et al. (2015) demonstrated the importance of a few QTLs with large effects controlling leaf Na concentrations and the K/Na ratio, despite their heritability being moderate. Leaf K and P concentrations were also negatively associated with leaf Na concentration. Breeding efforts have aimed to reduce leaf Na concentration by introducing a low leaf Na trait of *G. barbadense* into elite cultivars via backcrossing. Several high performing breeding lines with low leaf Na concentration were developed with outstanding yield and fiber quality properties (Liu S. et al., 2020). These results also support the neutrality of low leaf Na trait on agronomic performance but likely beneficial effect on nutrient use efficiency of K and P. The low leaf Na concentration was found to be associated with high Na in roots, therefore, the trait can drive Na redistribution so that more Na is sequestered in roots while maintaining low Na in leaf and plant canopy. Breeding efforts transferring the low leaf Na trait in elite cotton germplasm is ongoing.

## The Future Is Now!

Plant breeders alike are faced with the challenge of producing new cultivars that will perform under future production systems. While gains have and continue to be made through traditional plant breeding principles and techniques, modern breeding technologies continue to be developed and adopted in breeding programs. As such, commercial plant breeding operations require, and are ripe for transformative change. It is expected that when combined with traditional plant breeding, these novel techniques and importantly their integration, will improve selection accuracies and enable increases in the rate of genetic gain. Through the integration of GS, gene editing, phenomics and GM traits for yield enhancement with our existing largely traditional, field-based phenotypic breeding program, the CSIRO cotton breeding program is focusing its efforts on transforming our breeding program to harness these technologies and meet the challenges of the 21st Century.

### Genomic Selection

Molecular markers are an essential tool for mapping, cloning and introgression of genes underlying agronomic traits. The efficacy of map-based gene identification and cloning depends on the resolution of genetic maps constructed using genome-wide markers (Zhu et al., 2017). We have initiated genome-wide identification of single nucleotide polymorphisms (SNPs) in cotton using transcriptomic data and complexity reduced DNA sequences before a whole genome cotton sequence was available (Zhu et al., 2014). A portion of the identified SNPs were used in generation of CottonSNP63K (Hulse-Kemp et al., 2015). We have used this SNP chip and whole genome resequencing in mapping and cloning of several cotton genes responsible for important agronomic traits, including disease resistance, as well as in genome-wide association studies and genomic prediction (Zhu et al., 2015, 2018, 2020; Ellis et al., 2016; Gapare et al., 2017; Liu X. et al., 2020; Wilson et al., 2021). Diagnostic SNP markers for multiple traits are now routinely used in the CSIRO’s cotton breeding program.

Genomic selection (GS) was proposed as a molecular based breeding strategy utilizing quantitative genetic methods to assist breeding (Meuwissen et al., 2001). At its core, GS requires a training population of thousands of plants with both genotype and phenotype data. Based on the training population, a high dimensional regression model (Wang et al., 2018) is used to study the genotype and phenotype relationship. Then on a test population with only genotype data available, the models built using the training population were used to predict the genomic-estimated breeding values (GEBV) of the target traits. One assumption behind GS is that the genome-wide marker set used in the study should be able to be in close linkage disequilibrium with quantitative trait loci (QTL), so that the accuracy prediction accuracies of breeding values can be acquired without knowing the exact position of QTL. Because of the advances in next generation DNA sequencing techniques, acquiring a high-density marker set has become feasible and cheaper than phenotyping, which promotes GS to be applied in various crop species including wheat (Poland et al., 2012; He et al., 2016) and rice (Spindel et al., 2015).

The application of GS on cotton is still under development. Only a few pilot studies have been focused on demonstrating that genomic prediction models can provide accurate prediction to fiber qualities based on experimental crosses (Islam et al., 2020; Liu Y.-H. et al., 2020). In CSIRO, we have conducted two studies using real breeding materials collected from the CSIRO cotton breeding program. Gapare et al. (2018) evaluated performance of five Bayesian regression models on a small historical data set of 215 breeding lines with phenotypes collected over multiple locations. They found that the prediction models provided good prediction accuracy for fiber length. Li et al. (2022) conducted genomic prediction analysis on a full range of fiber quality traits and yield related traits based on a larger data set of 1385 breeding lines. They concluded that (i) the prediction accuracies were in line with the heritability of the traits, (ii) inclusion of the pedigree information into the genomic prediction models helped improve the prediction accuracies, and (iii) the importance of training population design. They also highlighted the potential challenges of modeling of multiple year and trial data, and the importance of integrating environmental data such as meteorological data into the prediction models (Crossa et al., 2021).

The routine deployment of GS into the CSIRO cotton breeding program will require additional research. We will target the implementation of GS to the single plant selection (SPS) stage of the breeding pipeline (Figure 2). We believe that this will optimize the outcome of selection and minimize the use of resources by targeting a stage in the breeding pipeline where phenotype accuracy is lowest and genetic variability highest, ultimately providing the most impact to GS. The prediction accuracies of the genomic prediction models on different traits need to be carefully evaluated, and compared to their counterparts in phenotypic selection, to determine a list of traits where the GS might be preferable over the conventional phenotyping approach. Another important element in the deployment of GS is training population optimization. Evidence has shown that using material in the training population that is closely related to material in the test population could lead to better predictive performance.

Further development of our GS model aims to incorporate high-resolution climate data as additional co-factors to enhance the model’s predictive power, as well as evaluating novel methods for modeling gene–environmental interactions. Another interesting direction is to adopt a multiple trait predictive model (Cheng et al., 2018) to simultaneously analyze multiple correlated phenotyping data. An additional aspect of future work incorporating co-factors to enhance genomic prediction accuracies is omics-guided genomic selection (OGGS) or gene-based breeding (GBB) (Zhang et al., 2020). OGGS for breeding specific traits holds the potential to tie deep-domain knowledge and understanding of cotton fiber development to accelerate precision breeding. For crops like maize, such approaches are helping advancing breeding of complex traits (Schrag et al., 2018; Azodi et al., 2019). In cotton, specific focus of targeted GBB has also progressed in accuracy of prediction (Liu Y.-H. et al., 2020). In this approach a list of differentially expressed genes (DEG) were compiled that related to fiber length, and about 200 SNPs were detected. This bi-parental population study revealed high prediction accuracies (up to 0.8) for fiber length. At CSIRO we are exploring the feasibility of OGGS and GBB with a focus on specific traits, including use of gene-networks that provide insights into key controlling clusters of genes that work together to create the unique cell walls of cotton fiber (MacMillan et al., 2017).

Extracting DNA material from plants at the earliest stage possible is not a new concept. It has been demonstrated that accessing embryo tissue within a plant seed was achievable and could generate DNA quantity and quality compatible with downstream analysis in maize (Papazova et al., 2005) or barley (von Post et al., 2003). The current development of such techniques in the CSIRO cotton breeding program is focused on feasibility and scalability. It is important for the technique developed to be non-destructive and to allow for plant growth and development after DNA extraction from the embryo. Zheng et al. (2015) demonstrated that this can be achieved in cotton and ensured high viability of cotton seeds. For this technique to be operable in a high throughput setting such as cotton genomic selection, it should be paired with genotyping and provide opportunity for automation. Plant seed comes in a diversity of shapes and forms, the technical aspect of the methodology is being designed, calibrated, and tested for cotton seed. Automation of the process, via DArT laboratory robotics (Diversity Arrays Technology, Bruce, ACT, Australia), enables the reproducibly and quality required for reliable next generation sequencing analysis scale. Current results shows that automated cotton seed drilling can produce quality DNA on a scale required for the deployment of GS and trait introgression.

### Gene Editing

Gene editing is a genetic engineering technology that allows targeted deletions, insertions, and other sequence alterations to be made at specific locations in the genome (Paterson et al., 2012; Li et al., 2014; Chen et al., 2020). As the editing machinery is either not integrated or can be segregated away from the alterations, plants produced using gene editing in many jurisdictions are considered equivalent to natural or induced mutations, so are non-GM and are not regulated (although this varies from country to country). Provided the target gene is known, this allows precise gene knockouts or even amino acid substitutions in an enzyme, for example, to change enzyme kinetics or substrate specificity and alter the biochemical makeup of a plant. Changes in plant gene regulatory sequence can also alter expression levels or tissue specific expression of any particular gene, so the possibilities for remodeling the genetic architecture of crops are endless. OGGS and GBB prediction-based methodologies are likely to provide potential targets for gene editing. In a breeding context, it may be possible to edit multiple favorable alleles for different agronomic traits directly in elite material. It may also be possible to re-engineer an endogenous disease resistance gene in a commercial cultivar to be identical to an alternate form found in a wild relative with resistance. This process would circumvent the long and involved process of trait introgression for disease resistance.

We are applying gene editing in several ways in cotton, both to identify the underlying genes conferring resistance to several pests and pathogens (to allow for more robust protection of our Intellectual Property through patents and to aid marker assisted selection – see section “Host plant resistance to insects and arthropods”) but also to generate new disease resistance traits by modifying the natural resistance mechanisms already present in cotton. For instance, using gene editing, we have generated a library of mutants with mutation(s) in individual isoforms of the miR482 family, which are predicted to regulate ∼15% of cotton nucleotide binding leucine-rich repeat receptors (Shen E. et al., 2020). These are currently under evaluation for novel combinations that confer resistance to our key cotton pathogens. The practical implementation of gene editing would be applied to the CSIRO cotton breeding program either at the end of the breeding process in elite cultivars, or alternatively at the start of the breeding process in germplasm identified as parents.

As gene editing requires knowledge of the target gene, knowledge of gene pathways leading to desirable phenotypes of the traits of interest are vital when deploying gene editing. The CSIRO cotton breeding program has invested in understanding these genetic pathways in a range of cotton fiber development studies. This includes cellulosic structural properties such as cellulose microfibril crystalline and paracrystalline fractions (Martinez-Sanz et al., 2017). Driving fiber development is an extensive set of genes, many of which are cell wall related. Transcript analysis of fiber through development, between species and of different tissue types (Tuttle et al., 2015; MacMillan et al., 2017) is providing a rich mine of information not only about the genes but the pathways and networks involved in controlling cotton fiber quality properties. Mining such cotton fiber gene-networks provides insight into key controlling clusters of genes that work together to create the unique cell walls of cotton fiber. This information will be used in the development of fiber improvement strategies, not only gene editing, but also GS and GM.

### Phenomics

The success of the CSIRO cotton breeding program can in part be attributed to its ability to accurately collect and analyze large quantities of specific phenotype data obtained under commercially relevant cultivation and management conditions, at the scale required by a commercial breeding program. The phenotype data that the program collects is currently centered around fiber yield, commercially important fiber quality parameters and disease resistance. The selection of lines based on these phenotypes has served the breeding program well. However, some phenotypes such as disease resistance are based only on qualitative assessments and fiber quality is restricted to the physical characteristics of the fiber rather than its composition, which may limit the ability to genetically predict fiber traits. Seed fiber yield, the most important trait, is only a single value that is the outcome of a integration of a complex web of parameters (Conaty and Constable, 2020), which precludes facile genetic prediction. Therefore, we see the need for the measurement of additional “novel” phenotypes through crop phenomics that can quantify a range of traits that have previously been either too difficult to quantify, or to obtain at scale (Atkinson et al., 2018; Crossa et al., 2021). One promising avenue is the use of hyperspectral/multispectral/thermal/digital cameras to capture data from many environments and stages of crop development. This data can be used to measure additional commercially relevant agronomic traits of interest, either at scale, accuracy or cost that traditional phenotyping is unable to achieve, enabling new breeding strategies to target these new traits of interest. These complex traits include crop water use, crop biomass development and photosynthetic capacity and yield components. These traits have all been identified as pathways to future yield progress in the CSIRO cotton breeding program and the addition of these new phenotypes will provide for a more fine-tuned selection of individuals in the breeding process.

The major application of these new phenomics tools to the breeding process is through the use of multi-trait analyses which improve the prediction accuracies of genomic estimated breeding values when the genetic and residual correlations are considered in the modeling process (Crossa et al., 2021). Because these genomic models take multiple traits and environments into consideration, along with interactions, they may be used to identify and exploit the correlations between different variables and for the differentiation of various effects. The integration of multi-trait genomic models with environment data and their interactions are likely to improve the accuracy of genomic prediction models and enable more specific breeding strategies for defined environment landscapes. These models can also be applied to the prediction of genotype performance in untested environments based on traits measured in alternative tested environments, and the prediction of costly or difficult to measure traits across all environments of interest (Crossa et al., 2021).

The successful deployment of GS in a commercial breeding program will be underpinned by an overhaul of the scale and accuracy of phenotypic data being captured and analyzed (Bhat et al., 2016). Over the past decade, computer vision and machine learning have developed and approaches such as deep learning have revolutionized the amount of data which can be processed, going far beyond what humans can meaningfully understand (Krizhevsky et al., 2012; LeCun et al., 2015; Chai et al., 2021). The number of studies using deep learning to extract valuable information from crops and horticulture imagery or image-like data (e.g., LiDAR) is rapidly expanding, in particular around crop classification, weeds or disease detection, and fruit counting (for review, see Kamilaris and Prenafeta-Boldú, 2018). Recent examples also include several studies quantifying stomata in different species, and state-of-the-art cereals biomass prediction from LiDAR data (Fetter et al., 2019; Zhu et al., 2021; Pan et al., 2022).

In cotton, deep learning has already been applied to detecting seedlings (Jiang et al., 2019), flowers (Jiang et al., 2020), bolls (Xu et al., 2021), leaf lesions (Caldeira et al., 2021; Liang, 2021), segmenting roots in soil (Shen C. et al., 2020), and identifying seeds from different cultivars (Zhu et al., 2019; Wu et al., 2021). Whilst these studies are encouraging and highlight some of the cotton traits and characteristics which can benefit from deep learning approaches, the accuracy and scale of the methodologies developed so far are not compatible with, and practical enough for, a commercial breeding effort. In an effort to build solutions designed to be used in a large breeding program, we have recently developed HairNet (Rolland and Farazi, 2021; Rolland et al., 2021, 2022). HairNet is a deep learning computer vision tool which replaces the visual scoring by expert breeders of cotton leaf hairiness, a trait linked to fiber yield, economical value, and insect resistance (Figure 4). When scoring a mixed population of glasshouse and field plants, HairNet reaches an accuracy of 89% with one image per leaf, or 95% with several images per leaf (Rolland et al., 2022). This accuracy can be pushed to 100% when scoring leaves of glasshouse-grown plants only (Rolland et al., 2022). The high accuracy of HairNet across environments and seasons demonstrates the value of a method which is operator- and weather-independent, and which produces imagery that can be revisited through time or for other purposes. Further efforts are needed to broaden the scope of phenotypes being captured in the field and at scale, to inform GS and breeding decisions. The CSIRO cotton breeding program is currently focusing on quantifying soil-borne diseases in the field, with yield and radiation-use efficiency identified as additional high-value targets.

### Genetically Modified Traits for Yield Enhancement

The success of GM traits for herbicide and insect management in cotton is well established. While we expect continued progress in this area, GM traits for enhanced productivity have been difficult to achieve. One example of a successful commercial launch of this technology is Bayer’s DroughtGard hybrid corn expressing a bacterial stress tolerance gene (Adee et al., 2016). However, this GM trait is not a silver bullet and only helps mitigate some of the yield losses experienced by farmers during droughts. An example being pursued in cotton is to identify target genes for GM that could improve carbon fixation under heat stress. As photosynthetic carbon assimilation underpins crop yield (Long et al., 2015; Sharwood, 2017), improving photosynthetic capacity has been highlighted as an area of interest for cotton breeding and development (Conaty and Constable, 2020). A key focus in the field of crop photosynthetic thermotolerance is Rubisco, the enzyme responsible for catalyzing the carboxylation or carbon fixation reactions of photosynthesis. Screening is currently underway in the cotton breeding program to identify potential candidates for photosynthetic improvement among the cotton cultivars and wild species of *Gossypium*. This long-term research is currently screening for superior Rubisco catalytic properties including increased catalytic speed, improved affinity for CO_2_ and an elevated specificity for CO_2_ as opposed to O_2_ (Sharwood and Bange, 2019), as well as identifying Rubisco activases with greater resilience at high temperatures. To date, wild species with origins in hot, dry climates have been identified with improved photosynthetic properties under heat stress. Further research is underway to determine photosynthetic traits that confer heat and drought tolerance to photosynthetic processes. Opportunities may also exist to identify germplasm with traits that improve mesophyll conductance. However, mesophyll conductance is a difficult to measure, complex trait controlled by many genes and environmental factors (Evans, 2021; Lei et al., 2021). Due to the incompatibility between cotton cultivars and many distantly related *Gossypium* species, photosynthetic trait transfer is likely to rely on synthetic biology approaches, of which are being investigated (Sargent et al., 2022).

## Meeting the Challenges of the 21st Century

Farmers are arguably the primary customers of all commercial plant breeding programs. Therefore, the challenges facing plant breeding programs are directly aligned with those challenges faced by producers. Put simply, plant breeding must assist farmers to maintain profitability in the face of rising costs of production, climate change and diminishing land availability, as well as altered social license and expectations.

Historically, substantial progress has been made in cotton breeding using traditional breeding techniques, which have continued to evolve as once novel techniques (e.g., marker assisted selection and GM traits) became routine. While we expect continued progress using these tried and proven techniques, to meet the challenges facing cotton breeding in the 21st Century, there is an imperative to deploy new technologies to assist in accelerating the progress of plant breeding. To ensure research impact is achieved, these technologies must be incorporated into traditional phenotypic breeding programs, enabling an increase in “data-driven breeding” capability. We believe that our largely traditional phenotypic breeding program will be enhanced with the addition of modern data-driven breeding techniques that harness the value of complex datasets through integration using algorithmic approaches.

**FIGURE 8:**
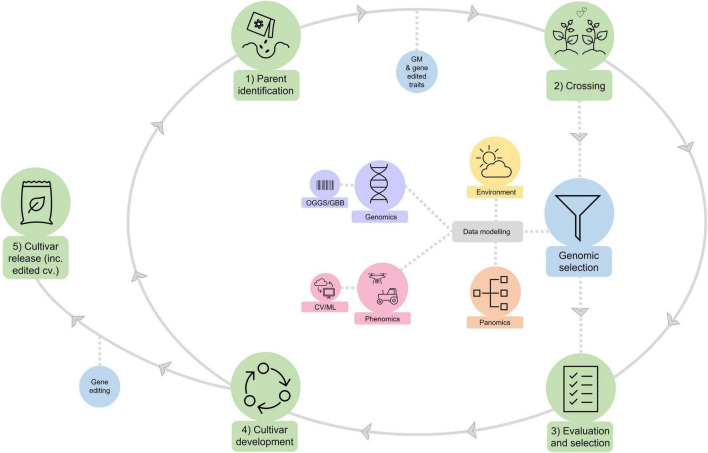
Pathfinder: A conceptual framework for how the CSIRO cotton breeding program aims to meet the challenges of the 21st Century. The traditional breeding operations of (1) Parent selection; (2) Crossing; (3) Evaluation and selection; (4) Cultivar release (all highlighted in green) form the foundation of the program. The CSIRO cotton breeding program will augment this traditional breeding process through the deployment of the new breeding technologies of genomic selection, genetically modified traits, and gene editing (highlighted in blue). Complex datasets encompassing genomics (including omics-guided genomic selection, OGGS, and gene-based breeding, GBB), season-long quantification of the plant (i.e., phenomics, including computer vision and machine learning, CV/ML, based phenomics, and panomics) and its environment will be integrated into genomic selection using algorithmic approaches.

With the rise of new breeding technologies and massive, complex datasets encompassing genetics, season-long quantification of the plant and its environment, some of the “art” of plant breeding is likely to be replaced by forms of artificial intelligence. The CSIRO approach to future cotton breeding is to use algorithmic approaches to integrate and prioritize the diverse streams of our research portfolio into the breeding process, that we call “Pathfinder” (Figure 8). Performance-based prediction algorithms and genotype data are the keystone of Pathfinder as they allow both the prediction of important traits via genetic estimated values, as well as the ability to determine which phenotypes and environmental variables are important to the prediction accuracy of the algorithm. Data from different plant phenotypes or environmental variables that improve prediction accuracy are prioritized over those that have minor or negative effects. Progress can be monitored by both changes in genetic gain and prediction accuracy for different traits over time. The remaining art of the breeder is in selecting both parents for crosses and progeny to find the right optimal settings for different traits to meet specific environments and market opportunities and to perform the necessary validation of the selected lines under commercial field and management environments.

Finally, it must be acknowledged that breeding technologies alone will not solve the challenges facing cotton breeding in the 21st Century. If these challenges are to be met, breeding programs must develop/maintain strong commercial relationships, a business development focus, long-term vision, and a willingness to test and adopt advantageous new technology combined with an equal willingness to drop technologies that provide no/minimal impact. The amalgam of these characteristics will ensure the challenges facing plant breeders in the 21st Century will be overcome.

## Author Contributions

KB, LE, XL, ZL, SL, DL, PM, VR, BR, DS, Q-HZ, and FP contributed knowledge and drafted sections of the manuscript. WC and WS conceived, contributed knowledge, and drafted the manuscript. All authors contributed and approved the submitted version.

## Conflict of Interest

BR is employed by Cotton Seed Distributors Ltd. The remaining authors declare that the research was conducted in the absence of any commercial or financial relationships that could be construed as a potential conflict of interest.

## Publisher’s Note

All claims expressed in this article are solely those of the authors and do not necessarily represent those of their affiliated organizations, or those of the publisher, the editors and the reviewers. Any product that may be evaluated in this article, or claim that may be made by its manufacturer, is not guaranteed or endorsed by the publisher.
